# Clinical Characteristics of Short-Stature Patients With Collagen Gene Mutation and the Therapeutic Response to rhGH

**DOI:** 10.3389/fendo.2022.820001

**Published:** 2022-02-16

**Authors:** Meiping Chen, Hui Miao, Hanting Liang, Xiaoan Ke, Hongbo Yang, Fengying Gong, Linjie Wang, Lian Duan, Shi Chen, Hui Pan, Huijuan Zhu

**Affiliations:** Key Laboratory of Endocrinology of National Health Commission, Department of Endocrinology, State Key Laboratory of Complex Severe and Rare Diseases Peking Union Medical College Hospital, Chinese Academy of Medical Science and Peking Union Medical College, Beijing, China

**Keywords:** short stature, skeletal abnormalities, collagenopathies, next-generation sequencing, growth hormone treatment

## Abstract

**Context:**

Clinical genetic evaluation has been demonstrated as an important tool to elucidate the causes of growth disorders. Genetic defects of collagen formation (the collagenopathies) have been reported to be associated with short stature and skeletal dysplasias. Etiological diagnosis of skeletal abnormality-related short stature is challenging, and less is known about recombinant human growth hormone (rhGH) therapy.

**Objective:**

This is a single-center cohort study which aims at exploring the genetic architecture of short-stature children with skeletal abnormalities and evaluating the frequency of collagenopathies to determine their phenotype, including the rhGH treatment response.

**Patients and Methods:**

One hundred and six children with short stature and skeletal abnormalities were enrolled who were evaluated by next-generation sequencing (NGS) to detect variants in the skeletal collagen genes including *COL1A1, COL1A2, COL2A1, COL9A1, COL9A2, COL9A3, COL10A1, COL11A1*, and *COL11A2*. The results were evaluated using American College of Medical Genetics and Genomics (ACMG) guidelines. Clinical characteristics and rhGH treatment response were summarized.

**Results:**

Twenty-four pathogenic or likely pathogenic variants of collagen genes were found in 26 of 106 (24.5%) short-stature patients with skeletal abnormalities, of which *COL2A1* mutations were the most common, accounting for about 57.7%. Other frequent mutations associated with skeletal development include *FGFR3*, *ACAN*, *NPR2*, *COMP*, and *FBN1* in 12.2%, 0.9%, 0.8%, 0.4%, and 0.4%, respectively, resulting in significantly different degrees of short stature. An overview of clinical features of collagenopathies showed growth retardation, skeletal abnormalities, and heterogeneous syndromic abnormalities involving facial, eye, hearing, and cardiac abnormalities. The average height of 9 patients who received rhGH treatment improved from a median of -3.2 ± 0.9 SDS to -2.2 ± 1.3 SDS after 2.8 ± 2.1 years. The most significant height improvement of 2.3 SDS and 1.7 SDS was also seen in two patients who had been treated for more than 6 years.

**Conclusions:**

A proband-based NGS revealed that distinct genetic architecture underlies short stature in varying degrees and clinical features. Skeletal abnormality-related short stature involving multiple systems should be tested for skeletal collagen gene mutation. Limited rhGH treatment data indicate an improved growth rate and height, and close monitoring of adverse reactions such as scoliosis is required.

## Introduction

Childhood linear growth is the result of chondrogenesis at the skeletal growth plate, the structure responsible for bone elongation and therefore overall body size (1). Recently, findings have uncovered a vast array of regulatory systems that implicate multiple aspects of the growth plate and long bone development and an accompanying vast array of genetic defects that can cause disorders of linear growth (2). Some sequence variations in genes affecting growth plate function can produce a phenotypic spectrum of short stature with skeletal dysplasia, ranging from severe skeletal deformity to disproportionately short stature, most of which show severe short stature (2). The etiological diagnosis for short stature with skeletal abnormalities is still a clinical challenge, and therapy for improving their severe short stature has been rarely attempted.

With the advances of broad sequencing approaches, clinical genetic evaluation has been demonstrated as an important tool to elucidate the causes of growth disorders from among the myriad possibilities, and an increasing number of short stature-associated genes have been discovered. These causative genes are involved in the physiological processes of the growth plate and long bone development, including normal production and action of multiple hormones, paracrine signaling, and extracellular matrix (ECM) molecules (e.g., cartilage oligomeric matrix protein, aggrecan, several different types of collagens produced by chondrocytes), as well as the normal function of multiple intracellular processes required for chondrocyte proliferation, hypertrophy, and extracellular matrix production (2, 3). Some typical genetic syndromes have been identified, such as Laron syndrome (MIM #262500) related to *GHR*, Leri–Weill dyschondrosteosis (MIM #127300) related to *SHOX*, Noonan syndrome (MIM #163950), and Silver–Russell syndrome (MIM #180860), which are associated with short stature and various multiorgan malformations (4–6). Genetic disorders associated with skeletal dysplasia include many genes involved in growth plate development, such as *FGFR3*, *ACAN*, *NPR2*, *FBN1*, and *IHH*, which can cause varying degrees of short stature with or without other minor abnormalities (7, 8). Recently, not only for *ACAN* (aggrecan) and *COMP* (cartilage oligomeric matrix protein) in ECM components but also for *NPR2* (natriuretic peptide receptor 2) in paracrine signaling, we reported the phenotypic and genotypic spectra and efficacy of GH therapy for height gain. For collagen, the most abundant protein in the human body, however, the prevalence of collagen gene mutation in short-stature patients with skeletal abnormalities is yet unknown, the current clinical manifestations of the disease are heterogeneous, and the response data of growth hormone therapy are limited. Collagen types II, IX, X, and XI are present in a growth plate important to long bone development and joint health, and type I collagen is the primary collagen in bone for bone formation, growth, and remodeling, and subsequently mineralization to form bone tissue. Mutations in genes that encode skeletal collagen are not uncommon in the genetic causes for growth defects with skeletal abnormalities (9).

Subsequently, we analyzed 106 children with short stature by using a gene panel for short stature and whole-exome sequencing (WES) from our single-center cohort and searched for variants in the skeletal collagen genes. Variant interpretation, genotype–phenotype analyses, and the response to rhGH treatment of skeletal abnormality-related short stature were investigated.

## Patients and Methods

### Patients

One hundred and six children with short stature and skeletal abnormalities in our endocrinology department were included, 64 of whom received WES and 42 of whom received a short stature-targeted gene panel sequencing. Skeletal abnormalities were characterized by an intrinsic abnormality in growth and (re-)modeling of cartilage and bone, including the whole-body skeleton of axial bones, limbs, and craniofacial bones, which were assessed by a professional physician through physical examination and measurements. All probands fulfilled the following diagnostic criteria: height standard deviation (SD) ≤ -2 with skeletal abnormalities, absence of abnormal findings on clinical examination or in laboratory tests that could account for short statures, such as hypothyroidism and GHD, and known Noonan syndrome and Turner syndrome. Clinical materials of the first and follow-up visits of the probands, including history-taking, physical examination, and auxiliary examination, were collected. Information about rhGH therapy was also reviewed and recorded. Peripheral blood samples of patients and their available relatives were collected, and genomic DNA was obtained from peripheral blood leukocytes by using standard techniques. The patients or guardians signed informed consent forms regarding the research, and this study was performed with the approval of the Ethics Committee of Peking Union Medical College Hospital.

### Whole-Exome Sequencing

The 3-μg genomic DNA concentrations were sheared with a Covaris LE220 ultrasonic instrument (MA, USA) to a target of 100–500-bp average size. Then, the DNA fragments with a main fragment size of 150–200 bp were screened by magnetic beads to create a DNA library for each subject. The library was qualitatively controlled by Agilent 2100 Bioanalyzer (BGI, Shenzhen, China). All amplified libraries were subsequently sent to BGI for circularization and sequencing on the BGISEQ-500 platform, and the primary sequencing data were read out. To detect the potential variants in the family, bioinformatics processing and data analysis were performed after receiving the primary sequencing data. Sequencing data were aligned to the human genome reference (hg19) using the BWA (Burrows–Wheeler Aligner) Multi-Vision software package to analyze single-nucleotide variants (SNVs) and INDEL calling (10). All SNVs and indels were filtered and estimated *via* multiple databases, including NCBI dbSNP, HapMap, 1000 human genome dataset, and database of 100 Chinese healthy adults.

### Targeted Sequencing

A capture panel (NimbleGen, Madison, USA) of short-stature genes was previously designed and assessed by our group. The capture panel covered all exons together with the flanking exon and intron boundaries (± 15 bp) of 466 genes. Sequencing was performed on an Illumina HiSeq 2500 or HiSeq 2000 platform in paired-end mode. In-house bioinformatic analysis was performed. The sequences were aligned to the reference human genome (HG19/HG20). The probe size was about 2.427 MB, and the theoretical capture efficiency of the probe was 98.83%.

### Data Analysis

To predict the effect of variants, we used *in silico* prediction programs to assess (PolyPhen-2, Mutation Taster, Provean, and scale-invariant feature transform [SIFT]). Pathogenic variants were under the protocol issued by American College of Medical Genetics and Genomics (ACMG) guidelines (11). The Human Gene Mutation Database (HGMD) was used to screen mutations reported in published studies. All the potential pathogenic variants observed by whole-exome and targeted panel sequencing as well as segregation analysis within family members were validated and genotyped by Sanger sequencing. Statistical analysis was performed using SPSS.25 software. All charts were completed in GraphPad Prism 8.0.2 software. Wilcoxon signed-rank test was used to explore the difference of height SDS in patients before and after rhGH treatment. The Kruskal–Wallis rank test was used to compare the height SDS and the height SDS changes after rhGH treatment in patients with collagen genes, *ACAN*, and *NPR2* mutations. p <0.05 was considered statistically significant.

## Results

### Genetic Architecture of Short Statue With Skeletal Abnormalities

**Figure 1:**
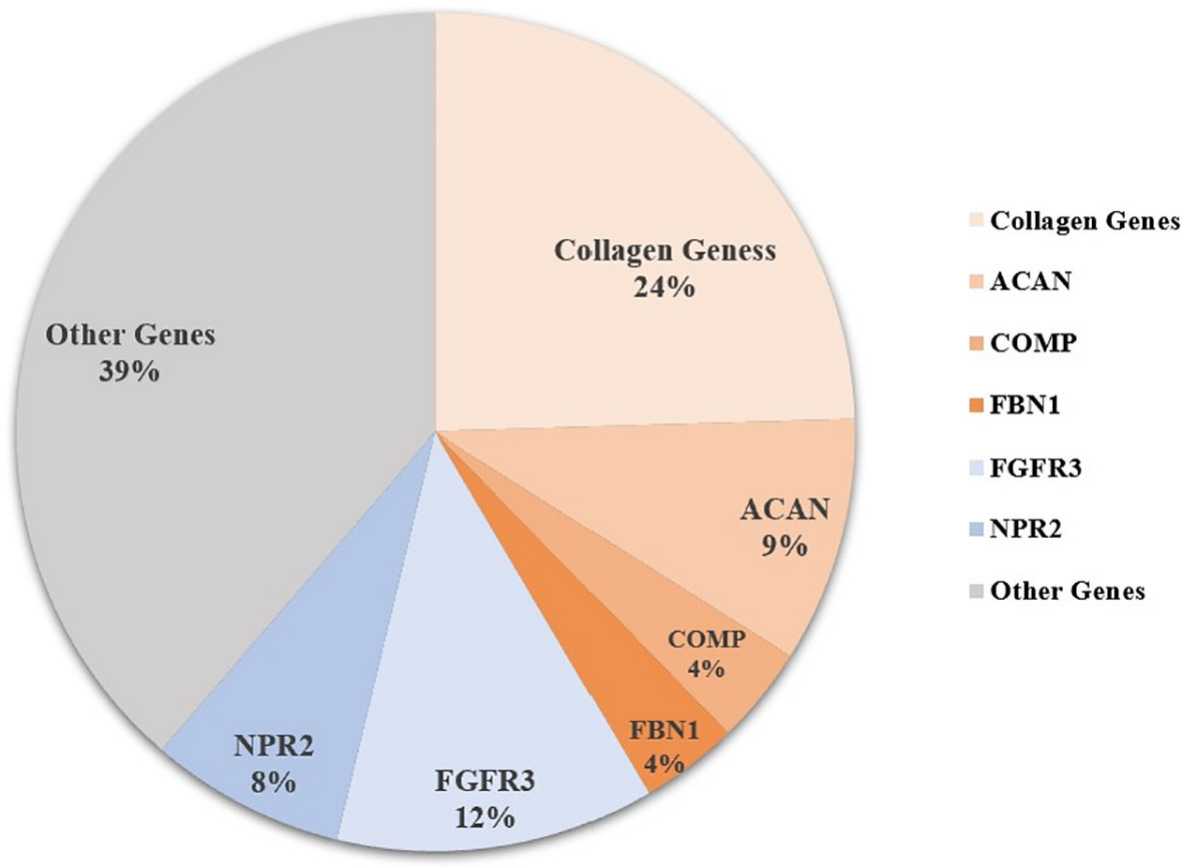
Genetic architecture of short statue with skeletal abnormalities from a single center. Other genes include *PTH1R, TRPV4, SHOX, KIF22, TRAPPC2, ARSL, RUNX2, CENPJ, FAM111A*, CNV (copy number variant), and some unknown causative genes.

Sixty-five patients were identified with genetic defects of cartilage extracellular matrix components in the 106 short-stature individuals with skeletal abnormalities, including 26 with collagen genes, 10 with *ACAN*, 4 with *COMP*, and 4 with fibrillin-1 (*FBN1*) mutation. Other frequent mutations in paracrine signaling of the growth plate development include fibroblast growth factor receptor 3 (*FGFR3*) covering 12.3% (13/106), *NPR2* covering 7.5% (8/106), and fibroblast growth factor receptor 3 (*PTH1R*) (n = 2). The remaining causal genes were associated with a fundamental cellular process, including *TRPV4* (n = 5), *SHOX* (n = 3), *KIF22* (n = 2), *TRAPPC2* (n = 1), *ARSL* (n = 1), *RUNX2* (n = 1), *CENPJ* (n = 1), *FAM111A* (n = 1), and pathogenic copy number variant (CNV) (n = 1) (**Figure 1** and **Table S1**).

Among this cohort, a total of 24 pathogenic or likely pathogenic rare variants in collagen genes were identified in 26 (24.5%) of the 106 short-stature individuals with skeletal abnormalities as per the ACMG guidelines, of which 5 variants were classified as pathogenic and 19 as likely pathogenic (**Table 1**). Type II collagenopathies were the most common. Fifteen patients, accounting for about 57.7%, had variants in the *COL2A1*, 3 had variants in the type IX collagen gene, 4 had variants in the type X collagen gene, and 2 had type XI collagen gene variants and 3 had variants in the type I collagen genes. Types of variant alleles include 18 (75.0%) missense mutations, 3 (12.5%) splicing mutations, 2 (8.2%) nonsense mutations, and 1 (4.2%) in-frame insertion mutation. Except for 4 biallelic heterozygous mutations, all the others were monoallelic heterozygous mutations. The results are summarized in **Table 1**. The mutations mainly occurred in the triple-helical region (17/24, 70.8%), followed by the C-terminal non-collagenous (NC1) domain (5/24; 20.8%) and the N-terminal non-collagenous (NC2) domain (2/24; 8.3%). Twenty-two of the variants are absent in public databases, whereas the c.1557+5C>T variant in *COL11A2* has been identified in 1/7,442 of East Asian chromosomes by the Exome Aggregation Consortium (ExAC, http://exac.broadinstitute.org), and the c.580G>A in *COL2A1* is present at an extremely low allelic frequency (4/140282) in gnomAD.

**Table 1:** In silico analysis and ACMG/AMP classification of collagen gene identified variants.

cDNA variant	Mutation status	Domain	Mutation taster	Polyphen-2	Provean	SIFT	ACMG-AMP classification
*COL2A1*							
c.196G>A(Asp66Asn)	Comhete.	NC2 domain	0.999/D	0.094/B	-0.83/N	0.17/N	Likely pathogenic
c.580G>A(p.Ala194Thr)	Heter.	NC2 domain	0.897/D	0.897/PD	-0.94/N	0.06/T	Likely pathogenic
c.1124G>T(p.Gly375Val)	Heter.	Triple-helical region	0.999/D	0.999/PD	-6.86/D	0.00/D	Likely pathogenic
c.1160G>A(p.Gly387Asp)	Heter.	Triple-helical region	0.999/D	0.999/PD	-5.33/D	0.00/D	Likely pathogenic
c.1202C>T (p.Pro401Leu)	Heter.	Triple-helical region	0.999/D	0.999/PD	-1.24/N	0.07/T	Likely pathogenic
c.1680+8_1680+9delGCinsTA	Heter.	Triple-helical region	NA	NA	NA	NA	Likely pathogenic
c.1789G>A(p.Gly597Arg)	Heter.	Triple-helical region	0.999/D	1.000/PD	-6.93/D	0.00/D	Likely pathogenic
c.2302-10C>T	Comhete.	Triple-helical region	NA	NA	NA	NA	Likely pathogenic
c.2401G>A(p.Gly801Ser)	Heter.	Triple-helical region	0.999/D	0.999/PD	-5.46/D	0.00/D	Likely pathogenic
c.2725G>A(p.Gly909Ser)	Heter.	Triple-helical region	0.999/D	0.999/PD	-5.07/D	0.00/D	Likely pathogenic
c.2965C>T(p.Arg989Cys)	Heter.	Triple-helical region	0.999/D	0.998	-7.09/D	0.00/D	Pathogenic
c.3472G>T(p.Gly1158Cys)	Heter.	Triple-helical region	0.999/D	1.000/PD	-8.41/D	0.00/D	Likely pathogenic
*COL9A1*							
c.2636C>A(p.Pro879His)	Heter.	Triple-helical region	0.999/D	0.997	-3.20/D	0.01/D	Likely pathogenic
COL9A2							
c.185C>T(p.Pro62Leu)	Heter.	Triple-helical region	0.999/D	0.05/B	-3.46/D	0.076/T	Likely pathogenic
c.1243G>C(p.Gly415Arg)	Heter.	Triple-helical region		1.000/PD	-6.67/D	0.00/D	Likely pathogenic
*COL10A1*							
c.1471C>T(p.Pro491Ser)	Heter.	Triple-helical region	0.999/D	0.509/D	-1.86/N	0.09/T	Likely pathogenic
c.1766T>G(p.Phe589Cys)	Heter.	NC1 domain	0.999/D	1.000/PD	-4.00/D	0.01/D	Likely pathogenic
c.1858_1865del CCTGTAAT (p.Pro620Valfs* 4)	Heter.	NC1 domain	1.000/D	NA	NA	NA	Pathogenic
COL11A1							
c.739G>T(p.Ala247Ser)	Heter.	NC1 domain	0.999/B	1.000/B	-0.44/N	0.80/T	Likely pathogenic
COL11A2							
c.1557+5C>T	Heter.	Triple-helical region	NA	NA	NA	NA	Likely pathogenic
*COL1A1*							
c.1386delT (p.Ala463Leufs*78)	Heter.	Triple-helical region	1.000/D	NA	NA	NA	Pathogenic
*COL1A2*							
c.2121_2122ins GCTGGTCCT (Pro707_Arg7 08insAlaGlyPro)	Comhete.	Triple-helical region	1.000/D	NA	NA	NA	Pathogenic
c.3583T>C(p.Cys1195Arg)	Comhete.	NC1 domain	0.999/D	1.000/PD	-10.31/D	0.00/D	Likely pathogenic
c.3997A>G(p.Thr1333Ala)	Heter.	NC1 domain	0.999/D	0.999/PD	-4.07/D	0.00/D	Likely pathogenic

Comhete., compound heterozygous; Heter, heterozygous; NC1, C-terminal non-collagenous; NC2, N-terminal non-collagenous; D, deleterious; B, benign; N, neutral; PD, possibly/probably damaging; T, tolerance; NA, not available.

### Clinical Phenotypes of Patients With Collagen Gene Mutation

The growth and phenotypic characteristics of individuals with collagen gene variations enrolled in the study are outlined in **Table 2**. The average age of patients with skeletal collagenopathies in this cohort was 7.4 ± 4.0 years for the 16 males and 10 females, and their bone age was consistent with chronological age with 6.9 ± 4.1 years, but we observed two type IX collagenopathy patients with a delayed bone age by about 3 years. The overall growth characteristics of patients with collagenopathies indicate growth retardation: four (33.3%) were born with small for gestational age (SGA); their average height Z-scores before rhGH treatment was -3.6 ± 1.4, and the calculated growth rate from 19 available individuals was 5.1 ± 1.7 cm/year. The average Z-score for weight and body mass index (BMI) was -1.3 ± 1.2 and 0.6 ± 1.4, respectively. Thirty-nine percent (9/23) was of familial short stature. The patients with proven collagenopathy and their affected parents had a median height Z score of -3.6 ± 1.3 and -4.1 ± 1.7, respectively.

**Table 2:** Baseline characteristics of individuals with skeletal collagenopathies.

	Type II collagen	Type IX collagen	Type X collagen	Type XI collagen	Type I collagen	Total
Demographic characteristics						
Male/female, n	9/6	2/0	3/1	1/1	1/2	16/10
Median age (n = 26)	7.6 ± 4.5	11.7 ± 2.2	7.5 ± 2.7	4.7 ± 0.1	5.8 ± 2.5	7.4 ± 3.9
Bone age (n)	7.3 ± 4.4 (11)	8.8 ± 2.8 (2)	6.7 ± 1.9 (4)	NA	5.7 ± 3.3 (3)	6.9 ± 4.1 (20)
Growth characteristics						
Growth velocity (n)	5.0 ± 1.5 (11)	5.8 ± 0.8 (2)	4.9 ± 2.5 (2)	4.8 ± 1.2 (2)	5.8 ± 2.3 (2)	5.1 ± 1.7 (19)
Height Z-scores (n = 26)	-4.1 ± 1.6	-3.5 ± 0.9	-2.7 ± 0.4	-3.2 ± 0.2	-2.6 ± 0.4	-3.6 ± 1.4
Weight Z-scores (n = 26)	-1.2 ± 1.4	-1.7 ± 50.7	-0.5 ± 0.8	-2.4 ± 0.7	-1.7 ± 0.7	-1.3 ± 1.2
BMI Z-scores (n = 26)	1.0 ± 1.2	-0.7 ± 0.8	1.6 ± 0.6	-0.8 ± 1.0	-0.8 ± 0.0	0.6 ± 1.4
IGF-1 Z-scores (n)	-0.43 ± 1.3 (10)	-5.1 ± 1.8 (2)	0.25 ± 1.8 (3)	-2.6 ± 1.1 (2)	-0.3 ± 1.2 (3)	-1.0 ± 2.1 (20)
SGA (n)	1 (11)	0 (1)	1 (2)	1 (1)	1 (3)	4 (18)
Family history (n)	6 (12)	0 (2)	2 (4)	0 (2)	1 (3)	9 (23)
Syndromic defects (n = 26)						
Midface hypoplasia	8	2	0	2	3	15
Thoracic deformity	6	0	1	1	0	8
Limb abnormalities	9	0	3	0	3	15
Scoliosis	8	1	4	0	1	14
Joint hypermobility	1	0	1	0	2	4
Congenital heart defect	0	1	0	0	2	3
Ocular abnormalities	1	0	0	1	1	3
Hearing loss	0	1	0	1	0	2
Cleft palate	2	0	0	1	0	3

BMI, body mass index; IGF-1, insulin-like growth factor I; SGA, small for gestational age; NA, not avaliable.

The main clinical manifestations are growth retardation, skeletal abnormalities, and heterogeneous syndromic abnormalities involving facial, eye, hearing, and cardiac abnormalities. All patients had skeletal abnormalities, among which limb abnormalities and spinal deformities are the most common, accounting for 57.7% and 53.8%, respectively. The main manifestations of bone involvement in skeletal collagenopathies include shorting and curving of long bone or phalanges of extremities, metaphyseal dysplasia of spine and limbs, arthrogryposis, joint laxity, scoliosis, or kyphosis. In addition, chest deformity, mainly presented as pectus carinatum, was also observed in 8 patients (30.8%). Fifty-eight percent of cases had facial abnormalities commonly observed in patients, mainly a low nasal bridge, high-arched palate, small jaw, or big or prominent ears. Cleft palate was observed in two patients with type II and one with type XI collagen gene mutations, and blue sclera was observed in two patients with type I and one with type IX collagen gene mutations. In addition, there were a small number of patients with other system defects, such as three with heart defects, three with congenital cataracts, strabismus, or amblyopia, and two with mixed deafness or ear deformity and congenital aural atresia.

### Phenotypic and Genotypic Analyses of Short Statue With Skeletal Abnormalities

Our data obtained from 15 probands with 12 kinds of *COL2A1* mutations showed that the phenotypic spectrum of *COL2A1* mutations included spinal deformity, abnormal cartilage development, midface hypoplasia, and ocular abnormalities. Almost all showed spine deformity including scoliosis, lordosis, dysplasia, and osteoporosis. The clinical manifestations of three boys (P.11, P.12, P.13 shown in **Table S2**) from three independent families with the same hot spot mutation (p.Arg989Cys) were consistent with spondyloepimetaphyseal dysplasia Strudwick type (SEDC) (MIM #184250), of which P.12 and P.13 had more severe skeletal manifestations and were diagnosed at 5.3 and 6.8 years of age, respectively, while P.11 was the milder type and diagnosed at a later age of 14.8 years. One novel missense variant (p.Ala194Thr) was identified and confirmed to segregate with the autosomal dominant short-stature phenotype in a family (P.3), which showed only short stature and flat round face with no obvious skeletal deformity in P.4. Besides, P.7 carries pathogenic variants in both *COL2A1* and *COL9A2* mutations resulting in a more severe skeletal deformity and short-trunk dwarfism, while the other two patients (P.16, P.17) in our study only carrying *COL9A1/COL9A2* mutation had very mild symptoms, with only mild chondrodysplasia.

In the data obtained from three probands in two independent families with *COL10A1* mutations, one missense variant 1766T>G (p.Phe589Cys) was *de novo* paternity and maternity confirmed in two identical twin brothers (P.18, P.19), and one truncating mutation c.1858_1865del CCTGTAAT (p.Pro620Valfs*4) was confirmed to segregate with the autosomal-dominant short-stature phenotype in five affected family members (P.21). Both types of mutations were located in the NC1 domain. All three probands and affected relatives exhibited typical metaphyseal chondrodysplasia type Schmid (SMCD) (MIN #156500) phenotypes with short bowed limbs, valgus knees, pronounced lumbar lordosis, posterior flexion of hips, enlarged large joints, and a faltering gait, which were consistent with radiographical findings. In addition, the proband with truncating mutations was born with flexion of the legs and had more severe forms of SMCD with additional manifestations such as short neck, pectus carinatum, beaded ribs, and widened epiphysis of the ribs. The two cases caused by missense variants exhibited relatively late-onset ages at around 2 years of age and mild or moderate manifestations.

Two novel variants in the type XI collagen gene were identified with one missense variant of *COL11A1* [c.739G>T(p.Ala247Ser)] and one splice site alteration of *COL11A2* (c.1557+5C>T). The girl (P.22) with the *COL11A1* mutation had a phenotype consistent with mild Marshall syndrome (MIM #154780), with midfacial hypoplasia, cleft palate, a less severe ocular presentation, but striking ocular hypertelorism, and short stature with spondyloepiphyseal dysplasia (12). The boy (P.23) with a *COL11A2* splice site alteration was characterized by Stickler syndrome (MIM #108300) with congenital cataract, sensorineural deafness, relatively short extremities with elbows valgus and joints pain, and typical midface hypoplasia.

Three patients with mutations in genes encoding type I collagen (*COL1A1* and *COL1A2*) were identified, and their skeletal abnormalities were characterized primarily by osteoporosis, with less common abnormalities in the limb bones and skull. In addition to skeletal abnormalities, they also have abnormalities in many organs, such as cardiovascular, joints, ligaments, midface development, and ocular anomalies. One patient (P.26) with biallelic heterozygous mutations [c.2121_2122insGCTGGTCCT (p.Pro707_Arg708insAlaGlyPro) and c.3583T>C (p. Cys1195Arg)] and one (P.24) with heterozygous truncating mutation [c.1386delT (p. Ala463Leufs*78)] had more severe osteogenesis imperfecta, such as early-onset motor retardation, heart defects, reduced thoracolumbar bone density, and obvious joint relaxation, than did the heterozygous missense mutations [c.3997A>G (p. Thr1333Ala)] (P.25).

The clinical phenotypes of other skeletal abnormality-related short stature were also briefly summarized based on our recent report (13–15). Heterozygous mutations in *ACAN* can lead to spondyloepiphyseal dysplasia, Kimberley type (MIM #608361), or osteochondritis dissecans (MIM #165800), which was consistent with the clinical findings of the 10 patients in our cohort, presenting as mild midface hypoplasia, short neck, thoracic deformity, spine malformation, short fingers/toes, short metacarpal bones, internal rotation of the elbow (contrast to cubitus valgus), and café-au-lait spots, and none of them complained of bone or joint pain. Four patients with *COMP* mutations exhibited typical pseudoachondroplasia (PSACH) (MIM #177170) with severe short-limb dwarfism, joint pain and stiffness, and early-onset osteoarthritis, and 4 patients with mutations in *FBN1* represented as acromelic dysplasia (MIM # 102370) shared severe short stature, short hands and feet, and joint limitations. Biallelic variations of *NPR2* mutation can cause acromesomelic dysplasia, Maroteaux type (AMDM) (MIM #602875), while monoallelic variants result in short stature with non-specific skeletal deformities and Miura-type osteochondral dysplasia. Autosomal dominant mutations in *FGFR3* causing achondroplasia (ACH) (MIM #100800) were found in 13 of our cohort. They appeared as short stature resulting from the shortening of the limbs with proximal segments affected disproportionally. In addition, the typical broad or protruding forehead, lumbar lordosis, and sacral kyphosis were seen in all these patients. Among the 8 cases of *NPR2* mutation in our study, except for one case with compound heterozygous mutation characterized by disproportionate short stature, mesomelic limb shortening, and shortened and broadened fingers and toes, conforming to AMDM, the other 7 cases were heterozygous mutation with or without disproportionate short stature, facial anomalies, and non-specific skeletal deformities, including mesomelic limb shortening, cubitus valgus, brachydactyly, shortened metacarpals or metatarsals, clinodactyly, and cone-shaped epiphysis.

### Comparison of Collagen Gene-Related Short Stature With Other Short-Stature Genes and Growth Response to rhGH Treatment

**Figure 2:**
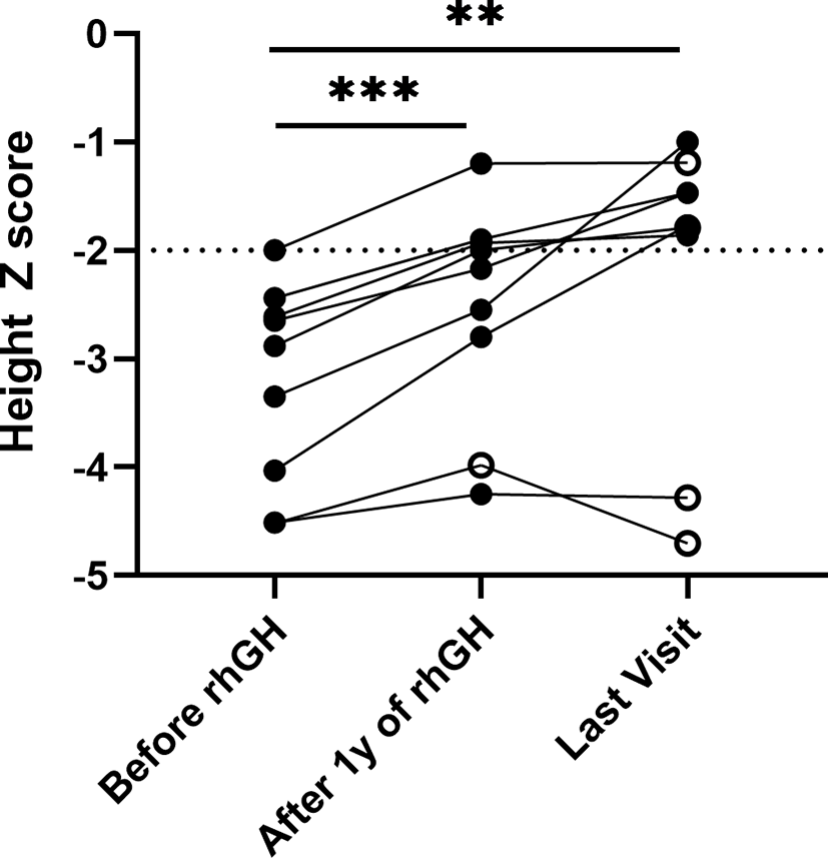
Height Z score in patients with collagenopathies with rhGH treatment. The median follow-up time was 2.25 years with a range of 1.12 to 6.50 years. Solid black dots indicate continued treatment up to the last follow-up, and hollow black dots indicate discontinuous treatment, **p <.01, ***p <.001.

There were 9 patients with collagen gene mutation who had received rhGH treatment, two of whom had combined treatment with gonadotropin-releasing hormone agonists (GnRHa) (**Table 3**). The initial age of treatment was 7.9 ± 3.5 years. After 1 year of treatment, the growth rate increased from 6.0 ± 1.6 to 9.0 ± 1.3 cm/years, and the average height Z score significantly increased from -3.2 ± 0.9 to -2.5 ± 1.0 (*p* <.01). Their average height Z score at the last follow-up was significantly increased to -2.2 ± 1.2 (*p* <.001) (**Figure 2**). The average duration of treatment was 2.9 ± 1.9 years, and three of them were treated discontinuously. Two cases (P.5, P.10) of scoliosis occurred after initial treatment, both of which discontinued the therapy for orthopedic evaluation (P.10 underwent spinal orthopedic treatment) and continued treatment. One girl (P.20) received rhGH at age of 5.7 and discontinued for personal reasons 1.3 years later, followed by the diagnosis of central precocious puberty with advanced bone age (+2~3 years), breast development at 7 years of age, and menophania at 9.8 years of age, and rhGH treatment was restarted combined with GnRHa for 1 year at 10 years of age, with poor therapeutic response. After exclusion of these three patients from growth response, the six remaining patients had an average improvement in a height Z score of 1.3 ± 0.5 (range, 0.8 to 2.3). The height of the two patients who had been treated for more than 6 years significantly increased their height Z score by 2.26 and 1.71, respectively. No abnormalities or side effects were observed throughout the treatment.

**Table 3:** Overview of patients with skeletal collagenopathies with rhGH treatment.

Patient ID	P.2	P.15	P.11	P.5	P.10	P.17	P.20	P.23	P.24
Mutation	*COL2A1*	*COL2A1*	*COL2A1*	*COL2A1*	*COL2A1*	*COL9A2*	*COL10A1*	*COL11A2*	*COL1A2*
Sex	F	F	F	M	M	M	M	M	M
Age of treatment (y)	11.25	11	3.75	13.67	4	10.08	5.67	4.58	7.33
Duration (year)	1.7^a^	1.25	6.00	0.2^b^ + 0.92^c^	1.17^b^ + 2^c^	2.83	1.25 + 1^a,c^	6.50	1.5
Growth velocity before (cm/year)	3.5	4.4	8	4.5	NA^d^	6.5	7.4	6	8
Height SDS before	-2.44	-2.65	-4.03	-4.51	-4.52	-2.61	-2.00	-3.35	-2.88
Growth velocity after 1 year	9.00	9.40	9	NA^d^	6.94	8.6	11.7	9.34	8.2
Height SDS at 1 year	-1.90	-2.17	-2.8	-4.25	-3.98	-1.93	-1.20	-2.55	-2.00
Height SDS change at 1 year	0.54	0.48	1.20	0.31	0.54	0.68	0.8	0.80	0.88
Last available height SDS	-1.47	-1.47	-1.77	-4.28	-4.7	-1.86	-1.19	-1.00	-1.79
Total height SDS change	0.97	0.97	2.26	0.23	-0.18	0.75	0.2	1.71	1.09

^a^Plus triptorelin.

^b^Accompanied by scoliosis during treatment.

^c^Discontinuous treatment.

^d^NA, not available.

**Figure 3:**
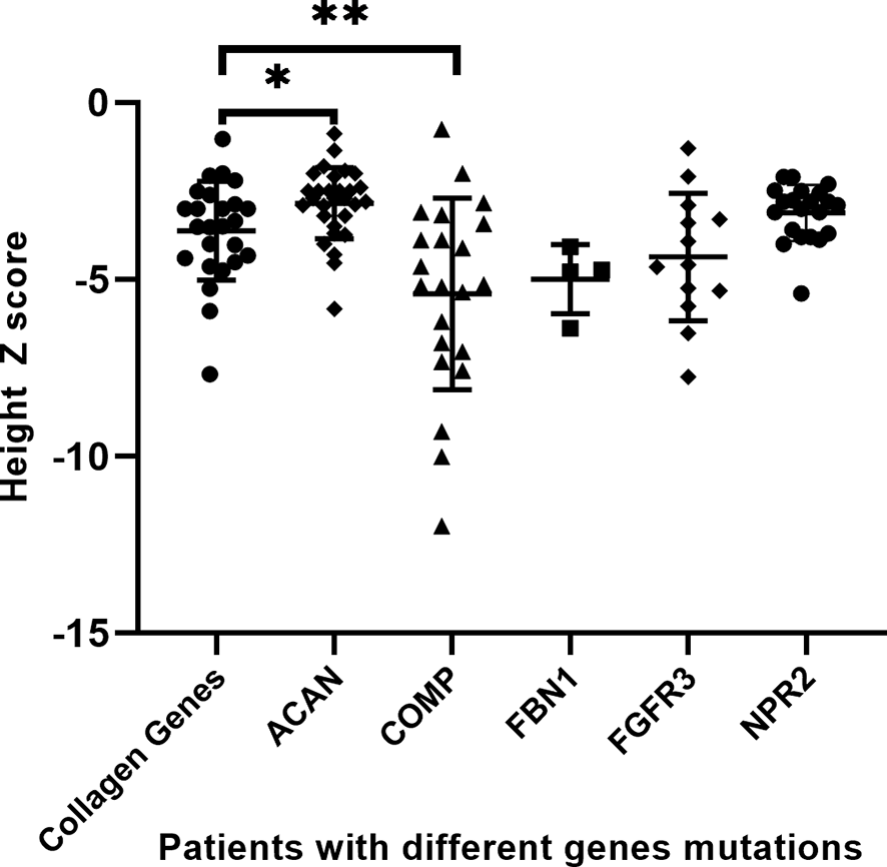
Comparison of different gene mutations leading to growth retardation. *p <.05, **p <.01.

We pooled demographic and treatment information of all short-stature patients with skeletal abnormalities both in our cohort and partly from the literature review, including other extracellular matrix component genes (*ACAN*, *COMP*, and *FBN1*) and paracrine signaling genes (*FGFR3* and *NPR2*), in which a subset of the 29 patients with *ACAN* mutation and 21 patients with *NPR2* mutation were from the literature review, based on previous reports (**Table 4**) **(** 13, 15). A total of 121 affected individuals diagnosed genetically are shown in **Table 4**. The gender proportion of males to females was 1.7 (73 males and 46 females). Their height Z score was −3.8 ± 2.0 from 114 affected individuals. The height Z score of males (70 cases) versus females (44 cases) was -3.9 ± 0.2 versus -3.7 ± 0.3 (*p* = .747), indicating that there is no difference in height between males and females. Six of the patients were adults with a height Z score of -6.8 ± 1.2, which was significantly lower than the 100 juvenile individuals with a height Z score of -3.7 ± 0.2 (*p* = .001), suggesting that the height impairment worsened with age and would be more severely affected in adults without any treatment. In addition, when the affected individuals were divided into extracellular matrix maintenance and paracrine signaling of the growth plate and long bone development according to physiological etiologies of short stature, there was no significant difference in their height impairment (*p* = .683). We compared the height Z score of collagenopathy patients with other different-causing gene mutations and found that the *ACAN* mutation resulted in milder short stature than the collagen gene mutation (*p* = .021), while the *COMP* mutation was the most severe (*p* = .009) (**Table 4** and **Figure 3**).

**Table 4:** Comparison of collagen gene-related short stature with other short stature genetic architecture.

		n	Age (year)	Sex		Height Z score		n	Treatment		Height Z score change	*p*
				Male	Female				Before	After		
Extracellular matrix	Collagen Genes	26	6.63 [3.67–10.25]	16	10	-3.62 ± 1.40	Collagen Genes	9	-3.22 ± 0.93	-2.53 ± 1.00	0.69 ± 0.28	**<0.001**
	*ACAN ^a^*	29	9.71 [5.53–12.2]	20	9	-2.85 ± 1.01*^d^*	*ACAN*	29	-2.85 ± 1.00	-2.22 ± 1.12	0.63 ± 0.71	**<0.001**
	*COMP ^b^*	27	5.60 [3.4–15.0]	15	10	-5.41 ± 2.71*^d^*	–					
	*FBN1*	4	5.83 [4.00–11.37]	2	2	-4.99 ± 0.98	–					
Paracrine signaling	*FGFR3*	13	5.83 [3.09–9.75]	5	8	-4.37 ± 1.80	*FGFR3*	4	-4.01 ± 2.27	-3.15 ± 1.47	0.86 ± 0.97	0.547
	*NPR2 ^c^*	21	7.00 [4.83–10.50]	14	7	-3.12 ± 0.79	*NPR2*	21	-3.12 ± 0.79	-1.98 ± 1.04	1.14 ± 0.68*^e^*	**<0.001**
Total		121	6.75 [4.00–11.17]	73	46	-3.75 ± 1.96	Total	63	-3.07 ± 1.05	-2.24 ± 1.11	0.82 ± 0.70	**<0.001**
						**<0.001**	***p***		0.438	0.427	**0.014**	

Data are expressed as median [interquartile range] and mean ± standard deviation (number of patients for whom the data were available).

Footnotes a–e indicate statistics within each group.

^a,b^Data were obtained from Liang et al. (13, 14).

^c^Data were obtained from Ke et al. (15).

^d^Significant (p <0.05 or less) vs. collagen genes.

^e^Significant (p <0.05 or less) vs. ACAN.

ACAN, aggrecan; COMP, cartilage oligomeric matrix protein; FBN1, fibrillin 1; FGFR3, fibroblast growth factor receptor 3; NPR2, natriuretic peptide receptor 2; SDS, standard deviation score.

The bold values mean p <0.05 or less.

**Figure 4:**
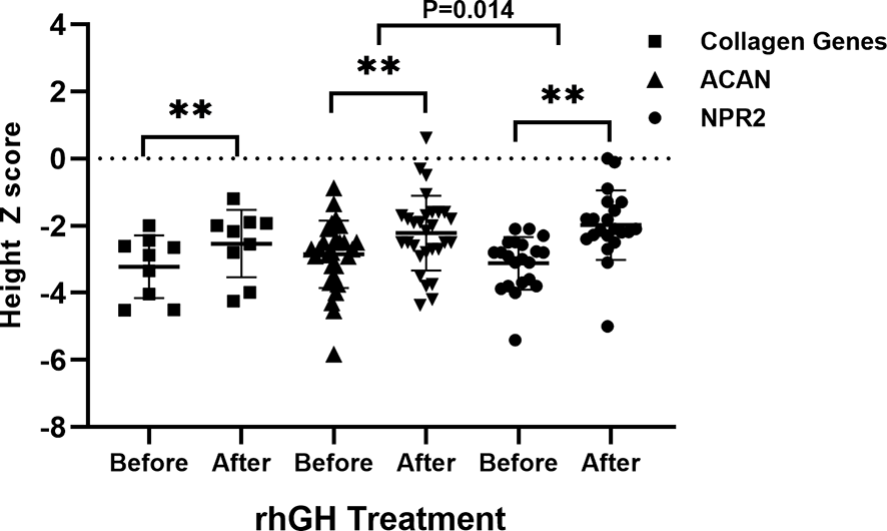
Comparison of growth response to rhGH between patients with collagen gene, *ACAN*, and *NPR2* mutations. **p <.01.

In short-stature patients with *ACAN* and *NPR2* mutation, rhGH had a significant effect on height gain as reported previously (**Figure 4**) (13, 15). In *ACAN*-related short stature, rhGH treatment significantly increased height and the height Z score (from -2.9 ± 1.0 to -2.2 ± 1.1) after 2.8 ± 0.4 years of administration. For NPR2-related short stature, height Z scores were significantly improved from -3.1 ± 0.8 to -2.0 ± 1.0 after 3.8 ± 0.6 years of treatment. Moreover, the growth response for rhGH treatment in *ACAN*-related short stature was better than *NPR2* (*p* = .014) (**Figure 4**). For *FGFR3*-related short, height Z scores were improved from -4.0 ± 2.3 to -3.2 ± 1.5, but this was not significant. However, given the severe PSACH phenotype caused by *COMP* mutations, limited benefit, and possibly serious complications, growth-promoting therapies were not recommended.

## Discussion

In this study, we performed NGS in short-stature patients with skeletal abnormalities and identified causal variants of skeletal collagen genes in 26 (24.5%) of the 106 individuals, among which 15 patients (14%) carried *COL2A1* mutations. Our molecular diagnosis rate of patients with skeletal abnormalities was 76.4% (81/106) and skeletal collagen genes accounted for 24.5% (26/106). *COL2A1* mutations were detected in 15 patients (14%), which was slightly higher than that of a recent NGS study on 82 short Chinese patients with clear signs of bone dysplasia (positive rate = 11%) (16). A recent study evaluating oligosymptomatic collagenopathies yielded a lower molecular diagnostic rate of 11.5% in 87 FSS patients treated with rhGH (17). Meanwhile, our study revealed the genetic architecture of short stature with skeletal abnormalities and proposed that mutations of collagen genes (especially *COL2A1*), *FGFR3*, *ACAN*, *NPR2*, *COMP*, and *FBN1* are common for short stature due to skeletal abnormalities in outpatient clinics in pediatric endocrinology. More importantly, we provided initial information about the phenotypical spectrum of collagenopathies by proving that short stature with skeletal abnormalities and heterogeneous syndromic abnormalities may be caused by mutations in the collagen genes.

Longitudinal growth of the skeleton is a result of endochondral ossification taking place in the epiphyseal growth plates of the long bones. The cartilaginous growth plate consists of extracellular matrix (ECM) and linear columns of differentiated chondrocytes that are organized into resting, proliferating, mature, and hypertrophic zones, which are continuously replaced by trabecular bone with the increase in length (1, 18, 19). Thus, multiple processes involved in the growth plate and long bone development, including basic cellular processes, extracellular matrix maintenance, paracrine signaling, and hormonal signaling, may underlie the distinct genetic architectures and physiological etiology of short stature with skeletal abnormalities. Collagens are a family of structurally related proteins that play a wide variety of roles in the ECM, are characterized by a basic structural coiled-coil right-handed triple helix, and are composed of three polypeptide chains (α chains). Both type II and X collagens are homotrimers composed of 3 identical chains encoded by the *COL2A1* and *COL10A1* genes, respectively. Both type IX and XI collagens are heterotrimers encoded by 3 different chains of *COL9A1*, *COL9A2*, *COL9A3* genes, and *COL11A1*, *COL11A2*, and *COL11A3*, respectively. Type I collagen is also a heterotrimeric molecule encoded by *COL1A1* and *COL1A2*. These genes encode procollagens, which are synthesized in the endoplasmic reticulum and contain a short N-telopeptide non-triple-helical (NC2) domain, a long triple-helical domain, and a short C-telopeptide non-triple-helical (NC1) domain, and then posttranslational modification generates mature collagens, which are secreted into the extracellular matrix (20, 21). In our study, of the 23 variants, 16 occurred in the triple-helical domain, highlighting the importance of this domain in the collagen genes. Consistent with previously reported results, all pathogenic variants of *COL10A1* in our study were in the NC1 domain, which contains motifs required for normal assembly of the collagen trimer (22). In addition, type II collagen fibrils have covalently linked type IX fibrils on their surface and at their core is a fibril template of type XI collagen. This association of types II, IX, and XI collagens can explain some of the phenotypic overlaps among the resulting conditions (23).

Since collagen is one of the components of many tissues and organs, genetic defects of collagen formation can affect almost every organ system and tissue in the body, and the clinical features often overlap, showing a variable syndrome in addition to bone phenotype. Patients with skeletal collagenopathies described in our study typically manifest growth retardation, skeletal abnormalities, and heterogeneous syndromic abnormalities involving facial, eye, hearing, and cardiac abnormalities, including osteogenesis imperfecta, a variety of chondrodysplasias, rarely, some forms of osteoporosis, osteoarthritis, joint hypermobility, and extra-skeletal features, for example, myopia, astigmatism, cataracts, sensorineural and conductive hearing loss, and mitral or tricuspid regurgitation. Other frequent gene mutations related to skeletal development, including *FGFR3*, *ACAN*, *NPR2*, *COMP*, and, *FBN1*, usually cause varying degrees of short stature, with or without other mild abnormalities, such as slight growth ratio imbalance and skeletal non-specific abnormalities (such as short finger/toe, short thumb, or midfacial dysplasia) (7, 8). In addition, one patient (P.6) with *COL2A1* mutation showed unexpected obesity and severe skeletal deformities and arthrogryposis, suggesting that weight gain and obesity may also be major concerns of epiphyseal dysplasia and contribute to the morbidity associated with joint problems. The underlying collagen mutation disrupts normal cartilage architecture, resulting in premature cartilage degeneration, and patients with these disorders often require joint replacement in the third to fourth decades of life (21). Both of our two patients with type IX collagen gene mutation showed a mild type of multiple epiphyseal dysplasias, which may be related to its high clinical heterogeneity and complex genetic background, and the late onset of the phenotype may also be one of the reasons as there was also a case that reported that symptoms did not appear until the age of 45 (24). Type X collagen is synthesized exclusively by hypertrophic chondrocytes in the cartilage growth plates of growing bones undergoing endochondral ossification, and its role of an extracellular scaffold or in the mineralization of hypertrophic growth plate has been proposed. Pathogenic variants in *COL10A1* cause reduced levels of functional type X collagen in the growth plate and contribute to the development of SMCD phenotypes, a disorder characterized by dwarfism and an expanded growth plate hypertrophic zone, which was seen in three of our patients with two novel missense and truncating variants (21). In the 2 patients with type XI collagen gene mutation, besides the typical skeletal and orofacial manifestations, P.22 with *COL11A1* missense mutation showed a less severe ocular presentation, while the boy with a *COL11A2* splice site alteration presented with obvious ocular anomalies of congenital cataracts. These findings are different from previous variants associated with Stickler syndrome caused by mutations in genes encoding type XI collagens, in which ocular anomalies are predominantly present in *COL11A1* mutation and the *COL11A2* heterozygous mutation usually causes non-ocular Stickler syndrome (25).

Although precise genotype–phenotypic correlations in collagen genes have not yet been established, we have made some interesting findings. Phenotypic severity might vary among patients with the same mutation, and in patients with *COL2A1* mutations, age at diagnosis might also be associated with disease severity, which was consistent with a previous study of *COL2A1* based on a large database (20). The genotype–phenotype correlation of *COL10A1* cases in this study was also consistent with previous reports (26, 27). Most of the identified mutations were present in the NC1 domain, which had motifs that control the formation of stable collagen X molecules by promoting the formation of the triple helix. The three cases of *COL10A1* mutation we reported all showed SMCD with short-limbed short stature, bowed legs, and a waddling gait, while two cases caused by missense variants exhibited relatively late-onset ages and moderate manifestations than the truncating one. More importantly, we compared the demographic and growth characteristics of patients with different disease-causing genes and found that children with skeletal dysplasia were usually severely short, and the heights of adults who did not receive treatment were significantly more impaired. In addition, among the short-stature individuals with causing genes, compared with individuals with collagen gene mutations, those with *ACAN* mutations showed a milder short stature, while PSACH with *COMP* mutations was at the severe end of the dwarfism spectrum and was associated with significant limb shortening (28). Overall, we still suggest that a larger phenotypic spectrum of collagen gene mutations would help construct a solid basis for further research of the genotype–phenotype correlation.

GH therapy has been introduced in several syndromic disorders with short stature, i.e., Noonan syndrome, Prader–Willi syndrome (PWS), and Silver–Russell syndrome (SRS), while there are limited data on the effect of rhGH treatment on children with short stature and skeletal dysplasia (29–31). Recently, we demonstrated a good effect of rhGH treatment in patients with *NPR2* heterozygous mutation, and the efficacy was negatively correlated with the initial age of treatment and was associated with gender and the gene positions of mutation (15). A study evaluating the efficiency of rhGH treatment with collagenopathy in a cohort also demonstrated a height Z score improvement from a median of –3.1 to –2.6 and to –2.2 after 1 and 3 years of therapy, respectively (17). Our study provides new evidence for the evaluation of rhGH therapy for skeletal collagenopathies. Consistent with previous results, the individual height Z score benefit after rhGH replacement varied considerably in this study (range: -0.18 to 2.26). During an average duration of 2.8 ± 2.1 years, the height Z score of 9 patients who received rhGH treatment improved from a median of -3.2 ± 0.9 to -2.2 ± 1.3 at the last follow-up, respectively. The most significant height Z score improvement of 2.3 and 1.7 was also seen in two patients who had been treated for more than 6 years. Limb and spinal deformities were a problem frequently seen in children with skeletal collagenopathies (57.7% and 53.8%). There was a concern about whether rhGH treatment would increase the frequency or severity of this finding, especially scoliosis. Two cases (P.5, P.10) of scoliosis occurred after initial treatment, both of which discontinued the therapy for orthopedic evaluation (P.10 underwent spinal orthopedic treatment) and continued treatment. This was also the main reason why rhGH is not recommended for patients with *COMP* mutations leading to PSACH, usually accompanied by severe osteoarthropathy. A previous clinical trial also demonstrated that in cases of PSACH, the height Z score was worse after rhGH therapy (32). Except for those two patients with the *COL2A1* mutation who developed scoliosis after initiating treatment, no exacerbation of scoliosis or other skeletal deformities was observed in the remaining patients. However, we do not think that the occurrence of this scoliosis was completely caused by rhGH treatment, because both patients carry hotspot mutation of *COL2A1* leading to SEDC, and the incidence of this disease is relatively high, which has been reported as 48% of 93 patients with molecularly confirmed SEDC or a related disorder in the previous literature (33). In addition, for the general short-stature population with skeletal dysplasia, *ACAN*-related short stature was more responsive to rhGH treatment than *NPR2*-related short stature, and significant height improvement was not seen in FGFR3-related short-stature patients in this cohort. While the effectiveness of rhGH for ACH caused by *FGFR3* mutation had been proposed in previous cohort studies (34), the very small sample size in our study may be the reason for the difference. A similar difference in rhGH efficacy was also observed in two patients with osteogenesis imperfecta patients who received bisphosphonates with or without rhGH. During the 2-year follow-up, the height Z score of P.24 combined with rhGH therapy was improved from -3.0 to -1.8, while the height Z score of patients treated with bisphosphonates alone was decreased from -2.0 to -3.3, which reveals the effectiveness of bisphosphonate combined with rhGH in patients with osteogenesis imperfecta. Moreover, there are quite a few studies that have confirmed the effectiveness of the combination therapy, not only in terms of growth velocity but also in bone mineral density and bone turnover (35–37). There were no complications such as fractures in both patients. Overall, this evidence suggests that rhGH treatment tolerability and efficacy in improving growth in patients with skeletal abnormalities vary greatly and careful consideration of indications for therapy and cautious observation during therapy are crucial for each patient.

Potential limitations of this work warrant consideration. First of all, in terms of molecular genetic testing approaches, WES may not detect large CNVs. Therefore, multiple ligation-dependent probe amplification (MLPA) or chromosomal microarray (CMA) analysis should be performed to screen for large deletions and insertions in genes belonging to the short-stature gene panel. In addition, some of the newly discovered variants still lack further validation, so we only evaluated their pathogenicity according to the ACMG guidelines, combined with their clinical phenotypes, pedigree verification, and *in silico* prediction programs. Furthermore, there are signs or clinical manifestations that have not been identified in some of our patients due to young age or too short follow-up. It is also worth mentioning that, unfortunately, we did not routinely assess bone mass in some children without a clear history of fractures. Moreover, some evidence for segregation of the variants with short stature within the families is lacking in some suspect pedigrees because of some unavailable relatives, which can strongly support the mutations’ pathogenicity. Lastly, more accurate genotype–phenotype correlations and evidence for the treatment of recombinant growth hormone deserve further study in a larger cohort of skeletal collagenopathies children with short stature.

In conclusion, skeletal collagenopathies are relatively frequent in syndromic-related short stature, and screening for collagen mutations should be considered in short-stature children with skeletal abnormalities. Although long-term studies evaluating rhGH treatment are insufficient and large cohort studies regarding rhGH dose, the optimal age to start treatment, and adverse events are lacking, initial information provided by our study about the efficacy of rhGH treatment for skeletal collagenopathies indicates an improved growth rate and height. Before starting rhGH treatment, patients with collagenopathy-related short stature should be extensively evaluated, and close monitoring of adverse reactions such as scoliosis is required.

## Data Availability Statement

The original contributions presented in the study are publicly available. This data can be found here: National Genomics Data Center (NGDC), PRJCA008063.

## Ethics Statement

The studies involving human participants were reviewed and approved by the Peking Union Medical College Hospital Ethics Committee. Written informed consent to participate in this study was provided by the participants’ legal guardian/next of kin.

## Author Contributions

MC and HZ designed the study. MC, HM, and HL collected the data. MC and XK conducted the data collection and analysis. FG guided the experimental study. MC and HZ drafted the manuscript. HY, LW, LD, SC, and HP interpreted the data and revised the manuscript. All authors contributed to the article and approved the submitted version.

## Funding

This study was supported by the CAMS Innovation Fund for Medical Sciences (CIFMS 2021-I2M-1-003).

## Conflict of Interest

The authors declare that the research was conducted in the absence of any commercial or financial relationships that could be construed as a potential conflict of interest.

## Publisher’s Note

All claims expressed in this article are solely those of the authors and do not necessarily represent those of their affiliated organizations, or those of the publisher, the editors and the reviewers. Any product that may be evaluated in this article, or claim that may be made by its manufacturer, is not guaranteed or endorsed by the publisher.
