# Corrigendum: Clinical Characteristics of Short-Stature Patients With Collagen Gene Mutation and the Therapeutic Response to rhGH

**DOI:** 10.3389/fendo.2022.896742

**Published:** 2022-04-06

**Authors:** Meiping Chen, Hui Miao, Hanting Liang, Xiaoan Ke, Hongbo Yang, Fengying Gong, Linjie Wang, Lian Duan, Shi Chen, Hui Pan, Huijuan Zhu

**Affiliations:** Key Laboratory of Endocrinology of National Health Commission, Department of Endocrinology, State Key Laboratory of Complex Severe and Rare Diseases Peking Union Medical College Hospital, Chinese Academy of Medical Science and Peking Union Medical College, Beijing, China

**Keywords:** short stature, skeletal abnormalities, collagenopathies, next-generation sequencing, growth hormone treatment

In the **Results**, subsection “*Comparison of Collagen Gene-Related Short Stature With Other Short Stature Genes and Growth Response to rhGH Treatment*”, paragraph 2 as published originally, **Table 4** was incorrectly cited. The following sentence “A total of 121 affected individuals diagnosed genetically are shown in **Table 3**.” should have read “A total of 121 affected individuals diagnosed genetically are shown in **Table 4**.”

Also, in the **Results**, subsection “*Comparison of Collagen Gene-Related Short Stature With Other Short Stature Genes and Growth Response to rhGH Treatment*”, final paragraph, incorrect height Z scores were presented.

The sentences “In ACAN-related short stature, rhGH treatment significantly increased height and the height Z score (from -2.9 ± 1.0 to 0.6 ± 0.7) after 2.8 ± 0.4 years of administration. For NPR2-related short stature, height Z scores were significantly improved from 3.1 ± 0.8 to 2.0 ± 1.0 after 3.8 ± 0.6 years of treatment.” should have read “In ACAN-related short stature, rhGH treatment significantly increased height and the height Z score (from -2.9 ± 1.0 to -2.2 ± 1.1) after 2.8 ± 0.4 years of administration. For NPR2-related short stature, height Z scores were significantly improved from -3.1 ± 0.8 to -2.0 ± 1.0 after 3.8 ± 0.6 years of treatment.”

The authors apologize for these errors and state that they do not change the scientific conclusions of the article in any way. The original article has been updated.

## Publisher’s Note

All claims expressed in this article are solely those of the authors and do not necessarily represent those of their affiliated organizations, or those of the publisher, the editors and the reviewers. Any product that may be evaluated in this article, or claim that may be made by its manufacturer, is not guaranteed or endorsed by the publisher.

