# Supplementary material for: Clinical Characteristics of Short-Stature Patients With Collagen Gene Mutation and the Therapeutic Response to rhGH
Source: Front Endocrinol (Lausanne). 2022 Feb 16;13:820001. doi: 10.3389/fendo.2022.820001 (PMC8889571; doi:10.3389/fendo.2022.820001)
Supplement: Supplementary file 1 [file Table_1.docx]

**Table S1. Summary of identified pathogenic/ likely pathogenic variants in non-collagen genes in patients with** **skeletal abnormalities.**

| **Patient ID** | **Gene** | **Gender** | **Age** | **cDNA** | **Protein** | **Mutation status** | **ACMG classification** |
| --- | --- | --- | --- | --- | --- | --- | --- |
| S.1 | ACAN | F | 4.7 | c.1411C>T | p.Gln471* | Heter. | P |
| S.2 | ACAN | M | 9.4 | c.1817C>A | p.Ala606Asp | Heter. | P |
| S.3 | ACAN | M | 9.8 | c.1762C>T | p.Gln588* | Heter. | P |
| S.4 | ACAN | M | 3.3 | c.2266G>C | p.Gly756Arg | Heter. | P |
| S.5 | ACAN | M | 14.6 | c.7469G>A | p.Cys2490Tyr | Heter. | P |
| S.6 | ACAN | M | 10.0 | c.1216G>A | p.Glu406Lys | Heter. | P |
| S.7 | ACAN | M | 5.5 | c.5211_5212delAinsGTTTCCTGACACT | p.Gln1738Phefs*19 | Heter. | P |
| S.8 | ACAN | M | 67.0 | c.1951delA | p.Arg651Glufs*52 | Heter. | P |
| S.9 | ACAN | F | 20.4 | c.1733-1G>A | - | Heter. | P |
| S.10 | ACAN | M | 14 | c.6970T>C | p.Trp2324Arg | Heter. | P |
| S.11 | FGFR3 | F | 3.7 | c.1138G>A | p.Gly380Arg | Heter. | P |
| S.12 | FGFR3 | F | 5.0 | c.1138G>A | p.Gly380Arg | Heter. | P |
| S.13 | FGFR3 | F | 2.7 | c.1620C>A | p.Asn540Lys | Heter. | P |
| S.14 | FGFR3 | M | 13.3 | c.1620C>A | p.Asn540Lys | Heter. | P |
| S.15 | FGFR3 | M | 2.8 | c.1620C>G | p.Asn540Lys | Heter. | P |
| S.16 | FGFR3 | F | 28.0 | c.1138G>A | p.Gly380Arg | Heter. | P |
| S.17 | FGFR3 | M | 8.0 | c.1138G>A | p.Gly380Arg | Heter. | P |
| S.18 | FGFR3 | F | 3.2 | c.1144G>A | p.Gly382Arg | Heter. | P |
| S.19 | FGFR3 | F | 6.7 | c.1144G>A | p.Gly382Arg | Heter. | P |
| S.20 | FGFR3 | F | 5.8 | c.1144G>A | p.Gly382Arg | Heter. | P |
| S.21 | FGFR3 | F | 10.0 | c.1626C>G | p.Asn542Lys | Heter. | P |
| S.22 | FGFR3 | M | 3.0 | c.1138G>A | p.Gly380Arg | Heter. | P |
| S.23 | FGFR3 | M | 9.5 | c.1620C>A | p.Asn540Lys | Heter. | P |
| S.24 | COMP | M | 10.8 | c.1393G>A | p.Gly465Ser | Heter. | P |
| S.25 | COMP | F | 24.0 | c.1552G>A | p.Asp518Asn | Heter. | P |
| S.26 | COMP | M | 9.7 | c.1417_1419del GAC | p.Asp473del | Heter. | P |
| S.27 | COMP | M | 5.7 | c.925G>A | p.Gly309Arg | Heter. | P |
| S.28 | FBN1 | M | 3.5 | c.5182G>A | p.Ala1728Thr | Heter. | P |
| S.29 | FBN1 | F | 5.5 | c.5174T >A | p.Ile1725Asn | Heter. | LP |
| S.30 | FBN1 | M | 11.83 | c.5243G>A | p.Cys1748Tyr | Heter. | LP |
| S.31 | FBN1 | F | 10.0 | c.5284G>A | p.Gly1762Ser | Heter. | LP |
| S.32 | NPR2 | F | 6.9 | c.2045C>T, c.2845C>T | p.A682V, p.R949* | Comhete. | P |
| S.33 | NPR2 | M | 13.4 | c.873+3A>G |  | Heter. | P |
| S.34 | NPR2 | F | 2.8 | c.2761C>T | p.Arg921* | Heter. | P |
| S.35 | NPR2 | M | 13.5 | c.833G>A | p.Arg278His | Heter. | P |
| S.36 | NPR2 | M | 4.5 | c.508T>C | p.Tyr170His | Heter. | P |
| S.37 | NPR2 | F | 13.0 | c.1576T>C | p.Ser526Pro | Heter. | P |
| S.38 | NPR2 | M | 2.9 | c.329delG | p.Arg110Profs*11 | Heter. | P |
| S.39 | NPR2 | M | 7.6 | c.329delG | p.Arg110Profs*11 | Heter. | P |
| S.40 | TRPV4 | M | 1.0 | c.2396C>T | p.Pro799Leu | Heter. | LP |
| S.41 | TRPV4 | F | 2.6 | c.2389G>A, c.760G>A | p.Glu797Lys, p.Val254Met | Comhete. | P |
| S.42 | TRPV4 | M | 8.0 | c.2389G>A | p.Glu797Lys | Heter. | P |
| S.43 | TRPV4 | F | 4.3 | C.2395C>G | p.P799A | Heter. | P |
| S.44 | PTH1R | F | 3.6 | c.251C>G | p.Ser84Cys | Heter. | LP |
| S.45 | PTH1R | M | 14.0 | c.629C>T | p.Ala210Val | Heter. | LP |
| S.46 | TRAPPC2 | M | 13.67 | c.271_275delCAAGA | p.Gln91Argfs*9 | Heter. | P |
| S.47 | ARSL | M | 2.0 | c.332G>A | p.Arg111His | Heter. | LP |
| S.48 | RUNX2 | M | 5.7 | c.577C>T | p.Arg193Ter | Heter. | LP |
| S.49 | CENPJ | F | 12.0 | c.2462C>T, c.3694G>A | p.Thr821Met, p.Gly1232Arg | Heter. | LP |
| S.50 | FAM111A | M | 3.0 | c.1085G>A | p.Trp362* | Heter. | P |
| S.51 | KIF22 | F | 32.0 | c.128G>A | p.Arg43His | Heter. | LP |
| S.52 | SHOX | F | 11.4 | c.350_351delAG | p.Arg118fs | Heter. | P |
| S.53 | SHOX | F | 13.5 | c.350_351delAG | p.Arg118fs | Heter. | P |
| S.54 | Dup | M | 8.7 | 46,XN,dup(15q14-q21.3).seq[GRCh37/hg19](40055963-57355379)*3 |  | Heter. | P |
| S.55 | SHOX | M | 10.0 | c.350_351AG | p.Arg118fs | Heter. | P |

Abbreviations: Comhete. = compound heterozygous, Heter = heterozygous, LP = Likely pathogenic, P = pathogenic, Dup = duplication.
