# Supplementary material for: Clinical Characteristics of Short-Stature Patients With Collagen Gene Mutation and the Therapeutic Response to rhGH
Source: Front Endocrinol (Lausanne). 2022 Feb 16;13:820001. doi: 10.3389/fendo.2022.820001 (PMC8889571; doi:10.3389/fendo.2022.820001)
Supplement: Supplementary file 2 [file Table_2.docx]

**Table S2. Summary of identified pathogenic/ likely pathogenic variants and phenotypes in patients with** **skeletal abnormalities.**

| **Patient ID** | **Gender** | **Age, yr** | **Gene** | **NM number** | **Mutation description** | **Mutation status** | **ACMG classification** | **Positive phenotypic features** |
| --- | --- | --- | --- | --- | --- | --- | --- | --- |
| P.1^a^ | F | 3.5 | *COL2A1* | NM_001844 | c.196G>A (Asp66Asn), c.2302-10C | Comhete. | LP | Micrognathia; scoliosis; salivation, motor development retardation. |
| P.2^a^ | M | 3.7 | *COL2A1* | NM_001844 | c.196G>A (Asp66Asn), c.2302-10C | Comhete. | LP | Micrognathia; scoliosis; salivation, motor development retardation. |
| P.3 | F | 9.8 | *COL2A1* | NM_001844 | c.580G>A (p.Ala194Thr) | Heter. | LP | Slightly blue sclera, almond eyes; elbow valgus, short 4^th^ and 5th metacarpals. |
| P.4 | F | 10.8 | *COL2A1* | NM_001844 | c.580G>A (p.Ala194Thr) | Heter. | LP | - |
| P.5 | M | 16.0 | *COL2A1* | NM_001844 | c.1124G>T (p.Gly375Val) | Heter. | LP | Prominent forehead, wide eye distance, low nose bridge; multiple skeletal deformities and arthrogryposis; osteoporosis of spine and pelvis, waddling gait; obesity, acanthosis nigricans, hyperlipidemia. |
| P.6 | F | 3.6 | *COL2A1* | NM_001844 | c.1160G>A (p.Gly387Asp) | Heter. | LP | Cleft palate, large head, prominent forehead, low nose bridge; valgus ribs, short limbs, valgus knees; scoliosis. |
| P.7 | M | 11.9 | *COL2A1/COL9A2* | NM_001844/  NM_001852 | c.1202C>T (p.Pro401Leu)/  c.1243G>C(p.Gly415Arg) | Heter./Heter. | LP/LP | Wide eye distance, epicanthus, low ear position, high-palate arch, inverted triangle face, multiple moles on the face, webbed neck; small hands, short fourth metacarpal bones, palms of both hands skewed toward the ulnar side, knuckle flexion, foot varus. |
| P.8 | M | 6.1 | *COL2A1* | NM_001844 | c.1680+8_1680+9delGCinsTA | Heter. | LP | Cleft palate, low nose bridge, bulbous nose, large ears; the fifth phalanx of both hands is short and curved; kidney cyst. |
| P.9 | F | 0.5 | *COL2A1* | NM_001844 | c.1789G>A (p.Gly597Arg) | Heter. | LP | Hip dysplasia; spinal dysplasia. |
| P.10 | F | 3.0 | *COL2A1* | NM_001844 | c.2401G>A (p.Gly801Ser) | Heter. | LP | Thoracic deformity, ribs valgus; lumbar lordosis. |
| P.11 | M | 13.8 | *COL2A1* | NM_001844 | c.2965C>T (p.Arg989Cys) | Heter. | P | Epicanthus, short neck; scoliosis. |
| P.12 | M | 6.8 | *COL2A1* | NM_001844 | c.2965C>T (p.Arg989Cys) | Heter. | LP | Strabismus, severe amblyopia; pectus carinatum; femoral epiphysis dysplasia; scoliosis. |
| P.13 | M | 5.3 | *COL2A1* | NM_001844 | c.2965C>T (p.Arg989Cys) | Heter. | P | High-arched palate, facial pigmented nevus, low bridge of nose, protruding ears; pectus carinatum, ribs valgus; knuckles hyperextension; lumbar lordosis. |
| P.14 | M | 11.2 | *COL2A1* | NM_001844 | c.2725G>A (p.Gly909Ser) | Heter. | P | Macrocephaly; pectus carinatum, rib valgus, thoracic deformity, beaded ribs; short long arms, spondyloepimetaphyseal dysplasia, lumbar lordosis. |
| P.15 | M | 6.5 | *COL2A1* | NM_001844 | c.3472G>T (p.Gly1158Cys) | Heter. | LP | Metaphysis dysplasia; Spinal dysplasia. |
| P.16 | M | 13.8 | *COL9A1* | NM_001851 | c.2636C>A (p.Pro879His) | Heter. | LP | Congenital malformation and atresia of the right ear. |
| P.17 | M | 9.5 | *COL9A2* | NM_001852 | c.185C＞T (p.Pro62Leu) | Heter. | LP | Hair weight at the back of the neck, blue sclera, high-arched palate; mild scoliosis; incomplete right bundle branch block. |
| P.18^b^ | M | 10.1 | *COL10A1* | NM_000493 | c.1766T>G (p.Phe589Cys) | Heter. | LP | Short limbs; multiple metaphysis dysplasia; mild lordosis. |
| P.19^b^ | M | 10.1 | *COL10A1* | NM_000493 | c.1766T>G (p.Phe589Cys) | Heter. | LP | Short limbs; multiple metaphysis dysplasia; mild lordosis. |
| P.20 | F | 3.7 | *COL10A1* | NM_000493 | c.1471C>T (p.Pro491Ser) | Heter. | LP | Short limbs; mild scoliosis. |
| P.21 | M | 6.3 | *COL10A1* | NM_000493 | c.1858_1865del CCTGTAAT (p.Pro620Valfs* 4) | Heter. | P | Pectus carinatum, rib metaphysis dysplsia; Short lower limbs, femoral bowing, genu varum, multiple epiphyseal and metaphysis dysplasia, bulged joints; lumbar lordosis. |
| P.22 | F | 4.8 | *COL11A1* | NM_080629 | c.739G＞T (p.Ala247Ser) | Heter. | LP | Cleft palate, ocular hypertelorism, flat nasal bridge, epicanthus. |
| P.23 | M | 4.6 | *COL11A2* | NM_080680 | c.1557+5C>T | Heter. | LP | High-arched palate, flapping ears, congenital cataract, conductive and sensorineural hearing loss (mainly sensorineural). |
| P.24 | M | 7.0 | *COL1A1* | NM_000088 | c.1386delT (p.Ala463Leufs*78) | Heter. | P | Blue sclera, astigmatism, poor vision, small jaw, facial pigmented nevus; tricuspid regurgitation with regurgitation. |
| P.25 | F | 8.1 | *COL1A2* | NM_000089 | c.3997A>G (p.Thr1333Ala) | Heter. | LP | SGA; saddle nose, high-arched palate, midface hypoplasia; short fingers and toes, curved phalanges, short and wide thumbs. |
| P.26 | F | 2.3 | *COL1A2* | NM_000089 | c.2121_2122ins GCTGGTCCT (Pro707_Arg 708insAlaGlyPro), c.3583T>C (p.Cys1195Arg) | Comhete. | LP | Prominent forehead, blue sclera, patent fontanelle; osteoporosis, loose joint ligaments of both hands; mitral and tricuspid regurgitation. |

Abbreviations: Comhete. = compound heterozygous, Heter = heterozygous, LP = Likely pathogenic, P = Pathogenic, ^a, b^ from the same family.
